# Neurological Complications in Young Infants With Acute Bacterial Meningitis

**DOI:** 10.3389/fneur.2018.00903

**Published:** 2018-10-24

**Authors:** Mei-Hsin Hsu, Jen-Fu Hsu, Hsuan-Chang Kuo, Mei-Yin Lai, Ming-Chou Chiang, Ying-Jui Lin, Hsuan-Rong Huang, Shih-Ming Chu, Ming-Horng Tsai

**Affiliations:** ^1^Division of Neurology and Pediatric Critical Care, Department of Pediatrics, Kaohsiung Chang Gung Memorial Hospital, Yunlin, Taiwan; ^2^College of Medicine, Chang Gung University, Taoyuan, Taiwan; ^3^Division of Pediatric Neonatology, Department of Pediatrics, Chang Gung Memorial Hospital, Taoyuan, Taiwan; ^4^Department of Nursing, Meiho University, Ping Tung, Taiwan; ^5^Division of Neonatology and Pediatric Hematology/Oncology, Department of Pediatrics, Chang Gung Memorial Hospital, Yunlin, Taiwan

**Keywords:** bacteremia, neurological complications, group B *streptococcus*, late-onset sepsis, meningitis

## Abstract

We aimed to evaluate the occurrence, treatment, and outcomes of neurological complications after bacterial meningitis in young infants. A case series study from a retrospective cohort from two tertiary-level medical centers in Taiwan between 2007 and 2016 was conducted. Eighty-five young infants aged < 90 days with bacterial meningitis were identified. 25 (29.4%) were born at preterm. Group B *Streptococcus* (GBS) and *Escherichia coli* caused 74.1% of identified cases. Despite the majority (90.6%) initially received microbiologically appropriate antibiotics, 65 (76.5%) had experienced at least one neurological complication identified at a median of 6 days (range: 1–173) after onset of bacterial meningitis. The most common neurological complication was seizure (58.8%), followed by subdural effusion (47.1%), ventriculomegaly (41.2%), subdural empyema (21.2%), hydrocephalus (18.8%), ventriculitis (15.3%), periventricular leukomalacia (11.8%), and encephalomalacia (10.6%). Nine patients (10.6%) died (including 4 had critical discharge on request) and 29/76 (38.2%) of the survivors had major neurological sequelae at discharge. Nighteen (22.4%) received surgical intervention due to these complications. After multivariate logistic regression, initial seizure (adjusted odds ratio [aOR]: 4.76, 95% confidence interval [CI]: 1.7–13.0, *P* = 0.002) and septic shock (aOR: 6.04; 95% CI: 1.35–27.0, *P* = 0.019) were independent predictors for final unfavorable outcomes.

**Conclusions:** Neurological complications and sequelae are common in young infants after bacterial meningitis. Patients presented with early seizure or septic shock can be an early predictor of final unfavorable outcomes and require close monitoring. Further research regarding how to improve clinical management and outcomes is warranted.

## Introduction

Bacterial meningitis is associated with high morbidity and mortality in neonates (1, 2). The predominant causative pathogens are Group B *Streptococcus* and *Escherichia coli*, causing 65–78% of all cases, with mortality rates varying from 13 to 25% in term-born and preterm infants (2–6). Studies of bacterial meningitis in adults have found that approximately three-fourths of patients have serious intracranial complications such as hydrocephalus, subdural empyema, infarction, and ventriculitis, which contribute to long-term neurological sequelae and final mortality (7–11). However, studies on the incidence and spectrum of complications and prognostic factors in young infants with bacterial meningitis are relatively scarce (12, 13).

Neurological complications after neonatal meningitis should be of more concern to physicians, because they can cause long term neurodevelopmental impairment and may require surgical intervention (7, 9). Furthermore, knowledge of the factors associated with poor prognoses could be valuable in selecting patients for more aggressive treatment strategies or at least intensive monitoring, in order to optimize the functional and normal behavioral abilities in neonates, who have had bacterial meningitis. In adult and pediatric settings, risk factors associated with neurological complications and sequelae have been identified, such as specific microorganisms, higher severity of illness, and early presentation of seizure and paresis (9, 10, 14–17); however, the conclusions of these studies are conflicting in young infants and only a few population-based cohort studies have been conducted. Therefore, we investigated the occurrence, treatment, and outcome of neurological complications in a large cohort from two medical centers in Taiwan with acute bacterial meningitis.

## Methods

### Patients, study design, and settings

All previously healthy patients younger than 3 months who presented to the Linkou and Kaohsiung Chang Gung Memorial Hospitals (CGMH) over a 10-year period (January 2007 to December 2016) with neurologic symptoms after bacterial meningitis were eligible for inclusion. Both Likou CGMH and Kaohsiung CGMH are tertiary-level medical centers in Taiwan with more than 10,000 pediatric patient hospitalizations each year. Linkou CGMH and Kaohsiung CGMH are the largest hospitals of north and south Taiwan, respectively. Cases were identified through the hospital meningitis registry and microbiology laboratory databases. All patients were followed for at least 1 year by the research team, mostly by the attending physician, who completed a case report form recording baseline variables of the patients and their evaluation after meningitis onset. Neonates with neurologic symptoms and sequelae attributable to a preexisting perinatal insults or neurologic disorder were excluded. This study was approved by the institutional review board of CGMH, and the informed consent was waived because all patient records and information were anonymized and de-identified prior to analysis.

### Clinical diagnostic criteria

Meningitis was defined in the presence of clinical signs of possible serious bacterial infection (defined based on World Health Organization) (18) and cerebrospinal fluid (CSF) culture positive for bacterial pathogens or blood culture/polymerase chain reaction (PCR)/latex agglutination positive for bacterial pathogens with a CSF leukocyte count > 20 × 10^6^/L. Episodes reported by physicians with negative CSF cultures were also be included if CSF results showed at least one individual marker of bacterial meningitis (defined as a glucose level of < 34 mg/dL [1.9 mlol/L], a ratio of CSF glucose to blood glucose of < 0.23, a protein level of more than 220 mg/dL, or a leukocyte count of more than 2,000/μL) (9, 19) and the clinical presentation was compatible with bacterial meningitis.

Neurological complications were defined as any newly neurological symptoms or signs and abnormalities on neuroimaging study [Transcranial ultrasound, computed tomography [CT] scan, or magnetic resonance imaging [MRI]] that occurred soon after an episode of meningitis, or judged by a clinical neonatologist to be directly resulted from an episode of meningitis. Neurological complications included:

1. Seizure: we clinically observed patients with any symptoms of subtle seizures, such as random or roving eye movements, sucking, unusual bicycling, or pedaling movements of the legs, etc. The electroencephalography (EEG) examination was arranged and regular anti-convulsants were given in patients with suspected seizure attack. The presence of seizure was defined as that observed clinically and/or abnormal epileptiform discharge recorded on the EEG without previous neurological or metabolic disorders.
2. Post-infectious encephalopathy: neonates who had changes in consciousness after stabilization of vital signs that lasted > 24 h after the onset of meningitis;
3. Hydrocephalus and/or ventriculomegaly: documented by transcranial ultrasound after the onset of meningitis, and in neonates without previous brain pathology;
4. The presence of any new focal infections, including subdural empyema, arachnoiditis, ventriculitis, and spinal or brain abscesses;
5. Other neurologic complications including neonates with encephalomalacia or cerebral infarction due to hypotension.

### Data collection and neurological follow up

Case-record forms were used to collect data on patients' demographics, symptoms and signs on admission, laboratory findings at admission and during hospitalization, clinical course, treatment and neurological findings at discharge, and final outcomes. After discharge, all surviving patients underwent a neurological examination performed by a neurologist, and the outcome was graded according to the validated Pediatric Version of the Glasgow Outcome Scale (GOS-E Peds) (20) A favorable outcome was defined as a GOS-E Peds score of 5 (good recovery) and an unfavorable outcome as a score of 1 (indicating death) to 4 (moderate disability). During the acute phase of these patients, transcranial sonography was routinely performed for all these patients. However, the brain CT or MRI would be done only when acute ill condition, clinically deteriorated situations, seizure attack, or when abnormal neurological findings were found. In the current study, all in-hospital mortality cases and those with critical discharge on request were considered as having unfavorable outcomes.

### Statistical analysis

Medians and proportions were used to describe continuous and categorical variables, respectively. The chi-square or Fisher's exact test was used to test for statistically significantly differences between categorical variables at the *p* < 0.05 level. Kaplan-Meier survival statistics were used to calculate cumulative rates of individual complications as well as the composite of any complications or death. Patients were censored at the time of death or at the end of the follow-up period. For predicting an unfavorable outcome at discharge, multivariate logistic regression was performed. The patients' demographics, initial clinical symptoms and laboratory results, and causative microorganisms were entered into a univariate logistic regression, and those with a *p*-value of < 0.1 were enrolled into the multivariate logistic regression model. All data were exported to Stata version 9.0 (Stata Corp., College Station, TX, USA) for analysis.

### Data availability

All data used for the analyses in this report are available and full anonymized data will be shared at the request from any qualified investigator.

## Results

### Clinical characteristics of young infants with acute bacterial meningitis

Eighty-five patients younger than 3 months of age with acute bacterial meningitis were identified. Twenty-five (29.4%) were born preterm (gestational age < 37 weeks). The mean gestational age and birth body weight were 37.8 ± 3.3 weeks and 2724 ± 541 g, respectively. A total of 44 (51.8%) episodes of bacterial meningitis were caused by group B *Streptococcus* (GBS), 19 (22.4%) by *E coli*, and 16 (18.8%) by other bacteria. A total of 6 patients had negative CSF cultures but at least one individual CSF marker and clinical symptoms of bacterial meningitis. The median age at diagnosis was 27 days (range, 3–180; Table 1). Eleven (12.9%) of them had a prenatal history of maternal fever and/or chorioamnionitis, and none of them had predisposing neurological disorders or perinatal insults.

**Table 1:** Epidemiological factors, co-morbid conditions, clinical data and laboratory parameters.

	**Alive and discharge with no neurological deterioration (*n* = 47)**	**Death and/or neurological deterioration (*n* = 38)**	***P*-value**
Age (days), median (IQR)	23.0 (7.5–65.0)	30.0 (18.8–60.0)	0.282
Gender (male/female)	24 (51.1)/23 (48.9)	17 (44.7)/21 (55.3)	0.562
Gestational age (weeks), mean ± SD	36.8 ± 3.5	36.9 ± 3.2	0.637
Prematurity (GA < 37 weeks)	14 (29.8)	11 (28.9)	0.908
Birth body weight (g), mean ± SD	2651.5 ± 417.2	2817.2 ± 467.4	0.264
Bacteria of meningitis			0.410
*Group B streptococcus*	22 (46.8)	22 (57.9)	
*E coli*	10 (21.3)	9 (23.7)	
*Pseudomonas spp*.	0 (0)	2 (5.3)	
Others	11 (23.4)	3 (7.9)	
CSF culture negative	4 (8.5)	2 (5.3)	
**CLINICAL PRESENTATION**			
Fever	38 (80.9)	31 (81.6)	0.932
Seizure (within 3 days of meningitis onset)	13 (27.7)	26 (68.4)	<0.001
Apnea, bradycardia and/or cyanosis	12 (25.5)	11 (28.9)	0.725
Feeding intolerance	14 (29.8)	21 (55.3)	0.018
Hypo or hyperglycemia	9 (19.1)	6 (15.8)	0.686
Respiratory failure (with intubation)	6 (12.8)	15 (39.5)	0.024
Electrolyte imbalance	14 (29.8)	19 (50.0)	0.057
Hypotension	3 (6.4)	13 (34.2)	0.002
Disseminated intravascular coagulopathy	4 (8.5)	11 (28.9)	0.014
Coagulopathy	7 (14.9)	16 (42.1)	0.007
Acute renal failure	1 (2.1)	2 (5.3)	0.584
Concurrent bacteremia	32 (68.1)	30 (78.9)	0.262
**LABORATORY PARAMETERS IN CSF [MEDIAN [IQR]]**			
CSF WBC at time of diagnosis (cells/mL^3^)	480.0 (76.0–2300.0)	400.0 (68.0–1546.3)	0.538
CSF protein at time of diagnosis (mg/dL)	287.6 (133.5–372.0)	283.9 (157.4–446.8)	0.456
CSF glucose at time of diagnosis (mg/dL)	33.5 (7.0–52.0)	28.5 (8.0–52.8)	0.704
**LABORATORY PARAMETERS [MEDIAN [IQR]]**			
WBC count (1,000/uL)	11.2 (3.9–15.9)	8.4 (3.2–14.4)	0.222
Hemoglobin (g/dL)	12.6 (10.1–13.5)	10.6 (8.9–12.2)	0.002
Platelet count (1,000/uL)	257.3 (132.3–413.2)	279.5 (181.2–373.5)	0.600
C-reactive protein (mg/L)	78.2 (39.2–139.2)	108.4 (10.4–208.8)	0.465

*CSF, cerebrospinal fluid; IQR, interquartile range, SD, standard deviation; WBC, white blood cell count*.

The demographics and clinical presentations of these patients are summarized in Table 1. Sixty-two (72.9%) had concurrent bacteremia; all of their blood cultures grew the same pathogen as those in the CSF culture. The majority of these symptoms were typical of neonatal sepsis, including temperature instability (81.2%), respiratory distress and/or apnea (27.1%), and feeding intolerance (41.2%). Seizure was present in 27 patients (31.8%) on admission. Sixteen (18.8%) had septic shock soon or within 24 h after admission, and 18 (21.2%) required intubation and ventilator support. Lumbar puncture was performed on admission in all patients. Thirty-four patients deteriorated clinically within 12 h after administration of empiric antibiotics and initial lumbar puncture, but none of them had transtentorial cerebral herniation with pupil dilation or abnormal posturing. The deterioration consisted of septic shock in 16 patients, respiratory failure in 14, new onset of seizure in 8 and a decreased level of consciousness in 4. All patients had a cranial sonography examination following bacterial meningitis, and cranial imaging was performed in 53 (62.4%) of 85 patients, mostly due to seizure and lethargy or irritability.

### Neurological complications

**Figure 1:**
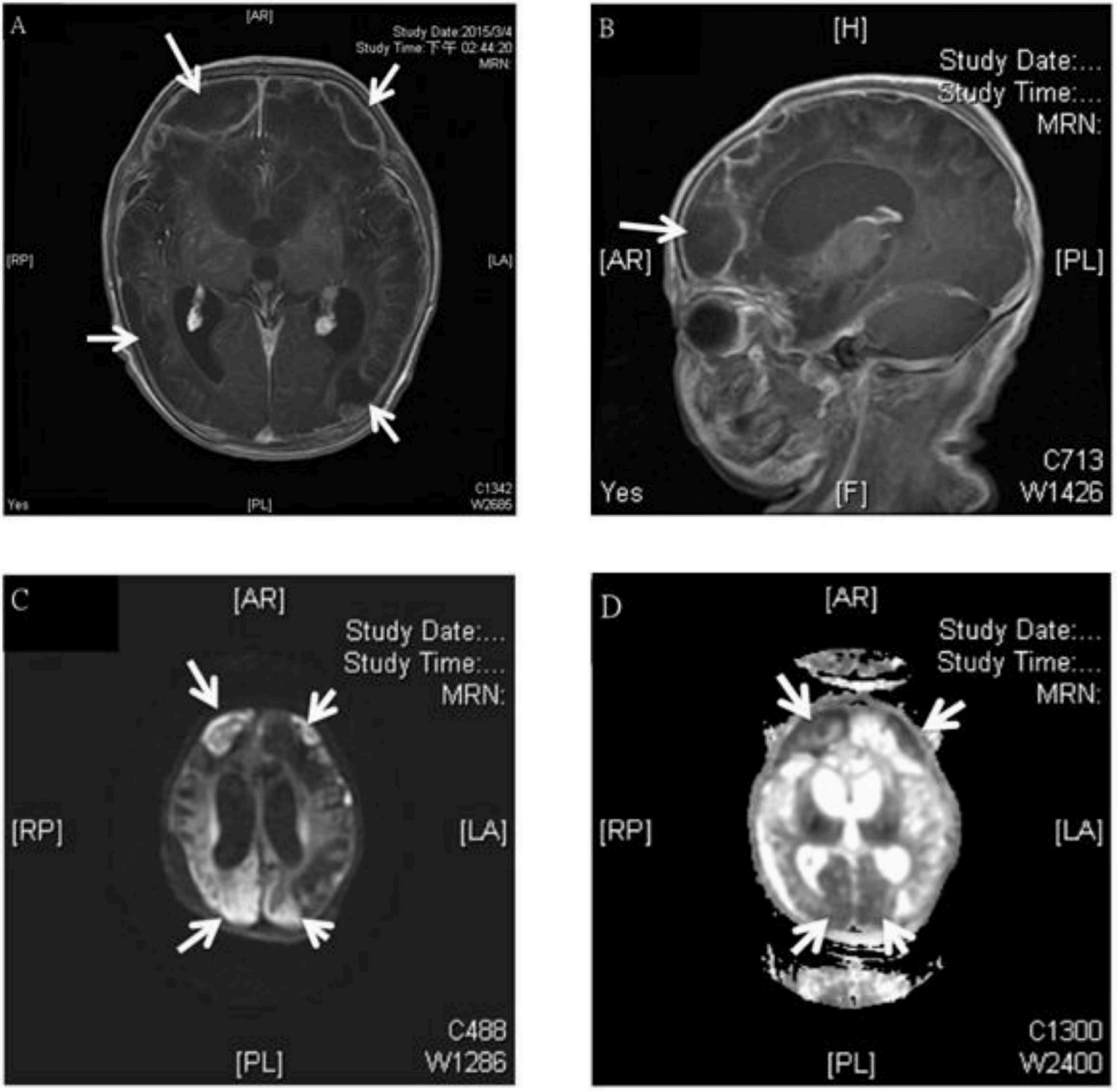
MRI study of a 3 week old boy with GBS subdural empyema. Axial **(A)** and sagittal **(B)** T1-weighted gadolinium-enhanced MRI showed subdural empyema over bilateral frontal, parietal, occipital area (arrows); diffusion-weighted **(C)**, and apparent diffusion coefficient **(D)**-weighted MRI of a subdural empyema over bilateral frontal, parietal, occipital area (arrows).

Neurological complications developed during clinical course in 65 of 85 patients (76.5%; Table 2). The most common neurological complication was seizure (50, 58.8%), followed by subdural effusion (40, 47.1%), ventriculomegaly (35, 41.2%), subdural empyema (18, 21.2%; Figure 1), and hydrocephalus (16, 18.8%). The occurrence of neurological complications was not associated with the causative microorganism, initial adequate or inadequate antibiotic treatment, or with any patient demographics. Among these neurological complications, subdural empyema, encephalomalacia, hydrocephalus, ventriculomegaly, increased intracranial pressure, and the presence of seizure were associated with a significantly higher rate of major neurological sequelae at discharge (Table 2).

**Table 2:** Neurological complications in young infants with acute bacterial meningitis.

	**Alive and discharge with no neurological deterioration (*n* = 47)**	**Death and/or neurological deterioration (*n* = 38)**	***P*-value**
Any neurological complications	29 (61.7)	36 (94.7)	<0.001
Seizure	18 (38.3)	29 (76.3)	<0.001
Subdural effusion	18 (38.3)	22 (57.9)	0.072
Increased intracranial pressure	7 (14.9)	17 (44.7)	<0.001
Ventriculomegaly	12 (25.5)	23 (60.5)	0.001
Hydrocephalus	3 (6.4)	13 (34.2)	0.001
Encephalomalacia	1 (2.1)	8 (21.1)	0.009
Subependymal hemorrhage	5 (10.6)	3 (7.9)	0.726
Ventriculitis	5 (10.6)	8 (21.1)	0.153
Periventricular leukomalacia	6 (12.8)	4 (10.5)	0.512
Infarction	2 (4.3)	6 (15.8)	0.075
Subdural empyema or abscess	5 (10.6)	13 (34.2)	0.015
Brain atrophy	0 (0)	2 (5.3)	0.197
Total antibiotic duration (days), median (IQR)	21.0 (17.0–28.0)	28.5 (21.0–45.8)	0.004

*IQR, interquartile range*.

**Figure 2:**
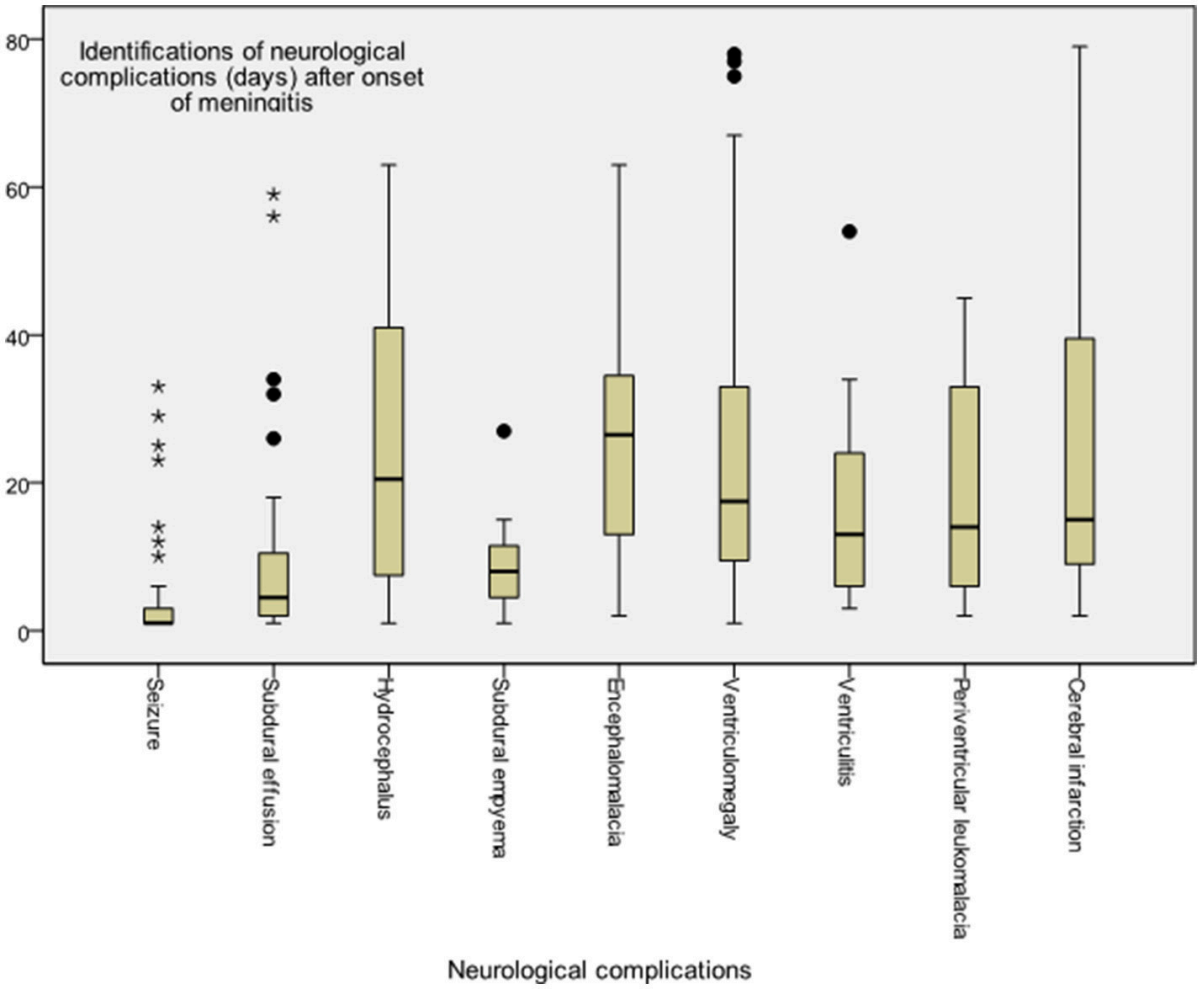
Time to diagnosis of various neurological complications in young infants with acute bacterial meningitis. Meningitis onset was defined as when the cerebrospinal fluid culture sampling was obtained, whereas onset of neurological complication was defined at the symptom presentation or diagnosis by neuroimaging studies.

**Figure 3:**
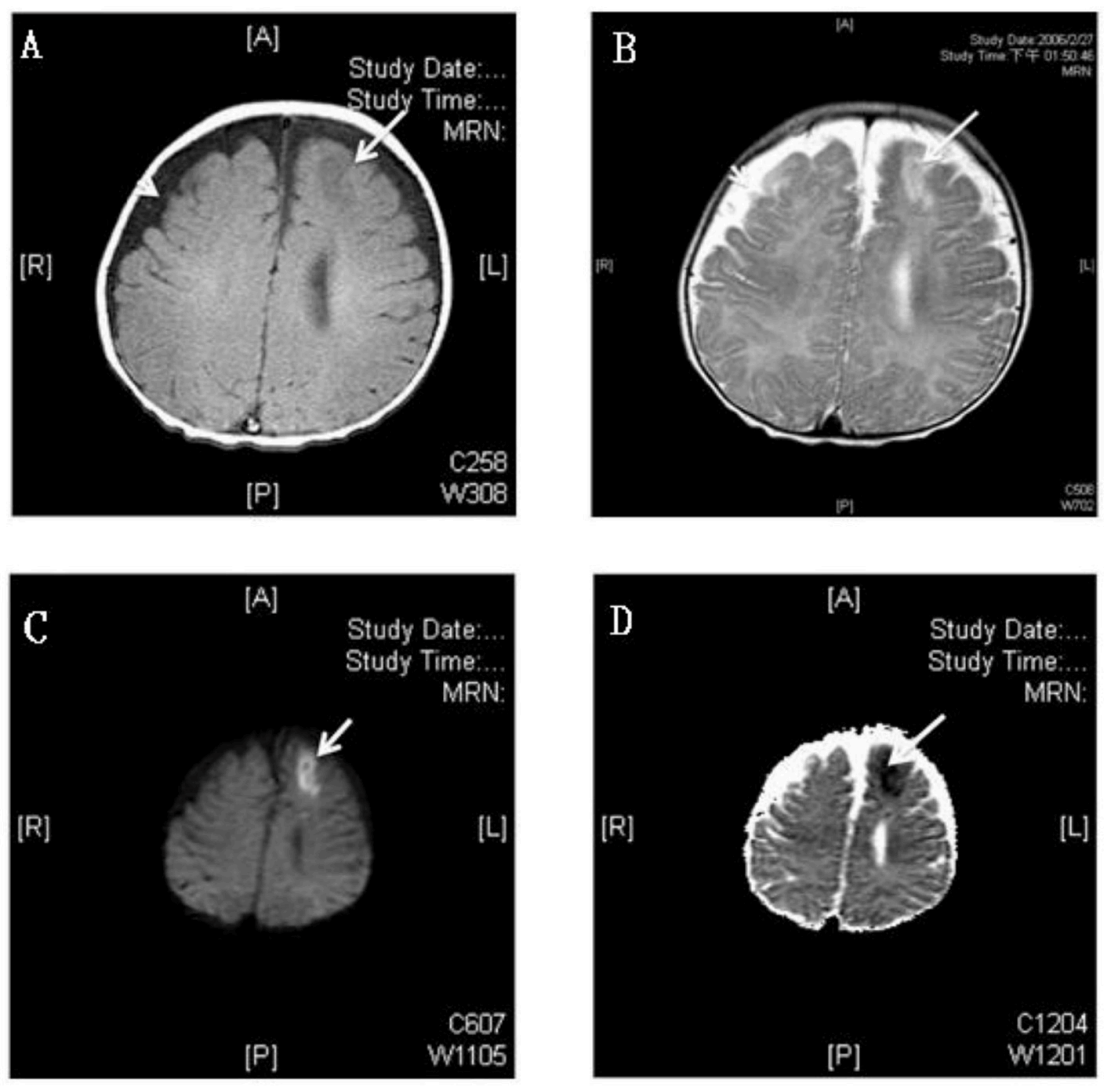
MRI study of a 3 month old boy with GBS meningitis and infarction. **(A)** Axial T1-weighted gadolinium-enhanced MRI showed hyperintensity and **(B)** T2-weighted MRI showed hypointensity over left anterior frontal area (white arrows) and bilateral frontotemporal subdural effusion (white arrow head); The left anterior frontal lesion showed hyperintensity over diffusion-weighted **(C)** and hypointensity over apparent diffusion coefficient **(D)** weighted MRI which revealed recent infarction (white arrows).

The time to diagnosis of all neurological complications from onset of meningitis (defined as the day of CSF culture taken) is summarized in Figure 2. The median time from the onset of sepsis to neurologic symptoms was 6 days [range, 0–169 days; interquartile range (IQR), 3–28 days], with different symptoms highly correlated with their onsets. Seizure accounted for the earliest onset of neurological symptoms (median time from sepsis, 1 days; range: 0–32 days), and encephalomalacia and cerebral infarction (Figure 3) were often the last to be detected (median time from onset of sepsis, 26 days; IQR, 19–39 days). Among the 50 neonates with seizure, seven (14%) were poorly controlled despite the administration of antiepileptic drugs for more than 48 h. Thirty-three (38.8%) patients had electrolyte imbalance, six developed central diabetes insipidus, and four had syndrome of inappropriate antidiuretic hormone secretion (SIADH).

### Treatment and outcomes

All of these patients received empiric antibiotics soon after blood culture was drawn; nearly two-thirds of patients received a combination of ampicillin/penicillin plus cefotaxime (61.2%, 52/85), with others receiving vancomycin plus cefotaxime (10.6%, 9/85) and vancomycin plus ceftriaxone (5.9%, 5/85). Seventy-seven (90.6%) patients received microbiologically adequate antibiotics within 24 h of symptom onset. However, the antibiotic treatment was modified in 70.6% (60/85) of patients, and the median duration of antimicrobial treatment in surviving patients was 22 days (range: 18–84 days).

Nineteen patients (22.4%) underwent surgical treatment: extraventricular drainage in 11 patients, subdural-peritoneal shunt in 2, ventriculoperitoneal shunt in 8 for treatment of hydrocephalus, and 8 had bilateral subdural drain. Seventeen of 19 patients who underwent surgical treatment survived, of whom 13 (76.5%) had neurological sequelae at discharge. Approximately one-third (6 patients, 31.6%) of these patients received repeated surgery, due to shunt infection in 3 patients and poor shunt function in 3. During the study period, the main treatment principles for these complications, including surgical intervention and follow-up protocols were similar.

Thirty-eight had an unfavorable outcome (44.7%; Table 2), including death in nine patients (10.6%), of whom four were critically discharged on request, four died of progressive multiple organ failure after meningitis, and one died of another episode of breakthrough bacteremia while still on antibiotic treatment. Twenty-nine of 76 survivors had neurological sequelae at discharge (38.2%). A significantly higher rate of unfavorable outcomes was observed in patients with neurological complications compared to those without any neurological complications (55.4 vs. 10.0%, *p* < 0.001). We aimed to find significant predictors of final unfavorable outcomes. We found initial septic shock, respiratory distress requiring intubation, electrolyte imbalance, and seizure within the first 3 days of meningitis were significantly associated with a higher rate of an unfavorable outcome (Table 3). After multivariate logistic regression, initial seizure (adjusted odds ratio [aOR]: 4.76, 95% confidence interval [CI]: 1.7–13.0, *p* = 0.002) and septic shock (aOR: 6.04; 95% CI: 1.35–27.0, *p* = 0.019) were the independent predictors for final unfavorable outcomes.

**Table 3:** Risk factors for final unfavorable outcomes (death or major neurological sequelae at discharge) by univariate and multivariate analysis.

**Parameters**	**Univariate analysis**		**Multivariate analysis**	
	**OR (95% CI)**	***P*-value**	**Adjusted OR (95% CI)**	***P*-value**
Preterm birth (GA < 37 weeks)	0.96 (0.38–2.46)	0.933	–	–
Seizure (within 3 days of meningitis onset)	5.67 (2.22–14.45)	<0.001	4.76 (1.74–13.02)	0.002
Septic shock	7.63 (1.98–29.36)	0.003	6.04 (1.35–27.04)	0.019
Respiratory failure (with intubation)	2.12 (0.88–6.61)	0.095	0.84 (0.21–3.39)	0.805
Electrolyte imbalance	2.36 (0.96–5.75)	0.059	1.41 (0.50–3.97)	0.516
High protein level in CSF (> median [250 mg/dL])	0.96 (0.39–2.37)	0.934	–	–
Low glucose level in CSF (< median [30 mg/dL])	1.13 (0.46–2.76)	0.797	–	–
Group B *Streptococcus*	1.50 (0.61–3.68)	0.376	–	–
Leukopenia (WBC count < 5,000 cells/uL)	1.33 (0.53–3.36)	0.543	–	–
Anemia (hemoglobin level < 11.0 mg/dL)	3.71 (1.48–9.26)	0.005	1.55 (0.51–4.75)	0.442
Thrombocytopenia (platelet count < 150,000/uL)	0.53 (0.17–1.55)	0.242	–	–

*GA, gestational age; CSF, cerebrospinal fluid; OR, odds ratio; 95% CI, 95% confidence interval*.

## Discussion

### General consideration of neurological complications after meningitis in young infants

The current study shows that neurological complications occurred in more than 75% of cases of bacterial meningitis in young infants and were associated with a high rate of unfavorable outcomes. The occurrence of neurological complications in young infants with meningitis was not associated with the causative pathogens, the appropriateness of initial antibiotics, or patients' demographics. Therefore, we suggest aggressively monitor the occurrence of neurological complications and arrange cranial imaging to detect patients with meningitis who develop neurological complications. After multivariate logistic regression, we found that initial seizure can significantly predict an unfavorable outcome (either death or neurological sequelae) at discharge.

A recent large national population-based cohort study found the incidence of bacterial meningitis in young infants to be around 0.38 per 1,000 live births and is associated with a significant case fatality rate of 8% (21). This surveillance study reported 23% of the survivors had serious central nervous system (CNS) complications (22). However, in the present study, we found a significantly higher rate of neurological complications in our cohort, and up to 44.7% of infants had neurological sequelae at discharge. We propose that different study populations, treatment policies and definitions of CNS complications may account for these different results. Further, different age groups, different causative pathogens, and underlying comorbidities may have different clinical presentations of bacterial meningitis and outcomes (17, 21–26). A recent review concluded that about half of survivors with bacterial meningitis suffered from focal neurological deficits (27); however, most of the studies in the review were conducted more than 10 years ago. When compared with reports from different age groups in recent years, we found neurological complications and sequelae, presented in 23–74% of all young infants (<3 months old) with bacterial meningitis (22, 28, 29), were significantly higher in young infants than children and adults (17, 21–25), except one study of patients with alcoholism found unfavorable outcomes in 58% of acute bacterial meningitis cases and 25% of patients died (30).

GBS remains the most common pathogen causing bacterial meningitis in young infants <3 months old (3, 31). Several studies have documented that intrapartum antibiotic prophylaxis in pregnant women with GBS colonization has lowered the incidence rate of GBS early-onset disease; however, this has no significant effect on GBS late-onset disease (32, 33). In our cohort, GBS accounted for more than half of bacterial meningitis cases, mostly presented as late-onset disease (33 cases, 84.6%) and our previous studies found that more than half of GBS invasive disease cases in the neonatal intensive care unit cause meningitis (34). Furthermore, GBS meningitis cases in the young infants were reported to have a mortality rate of 11.4% (3, 32), and 32% of GBS meningitis survivors have neurodevelopmental impairment (31). Although few invasive GBS strains were resistant to penicillin, which means rare GBS meningitis cases were treated with inadequate antibiotics, the neurological complications still happened despite timely effective antibiotic treatment (3, 32). Therefore, aggressively monitoring the occurrence of neurological complications in young infants with meningitis is certainly necessary.

### Prognostic factors of neurological complications

A recent large cohort study found temperature instability, seizure, higher CSF protein values, and pneumococcal meningitis were independently associated with serious CNS complications (22). We found seizure within 3 days after onset of meningitis as the independent predictor of final neurological sequelae, which can be explained by the evidence that more severe and acute brain parenchymal inflammation, an important contribution, and precipitation of seizure (35, 36), may cause more neurological damage. Therefore, we can identify neonates with initial seizure to be those who warrant early follow-up and intervention. For the association between higher CSF protein values and subsequent CNS complications, the results of previous studies have been somewhat conflicting (22, 29, 37, 38). However, previous studies have demonstrated that high protein levels in CSF are related to more severe inflammatory reactions and immune responses during bacterial meningitis (39, 40), which means more brain parenchymal damage. Therefore, it seems reasonable that high CSF protein levels can be a predictor of later poor outcomes, although this was not observed in the present cohort.

In the present cohort, subdural empyema or abscess occurred in approximately one-fifth of young infants with bacterial meningitis, which is a higher percentage than in previous reports (9, 27). Previous studies found that postmeningitic subdural empyema is more likely to occur in infancy due to the unique pathophysiology in infants (41, 42). In the present cohort, only nearly half (8, 44.4%) of patients underwent neurosurgical intervention for empyema, and none of them received surgical evacuation. Most patients received prolonged antibiotic treatment (a median duration of 45 days; range: 24–73 days) and only one patient finally died. Our experience is compatible with a recent guideline that 3–4 weeks of antibiotics is recommended if an empyema has been evacuated, and an optimal outcome can be achieved with longer duration if the patient is conservatively treated (9, 43).

The present study has several important limitations. First, we did not routinely perform regular cranial imaging follow-ups, and some neurological damage might have been missed, including asymptomatic subdural effusion and subclinical subdural empyemas. Second, most patients received lumbar puncture after initiation of empiric antibiotics and some of them had negative CSF cultures. These cases were enrolled only if the attending physicians registered in the database, and therefore culture-negative patients were underrepresented. Furthermore, some of the treatment practices and antibiotic choices may have changed during this 10-year study period. Thus, there is a little bit limitation of reliability in the conclusion. Finally, we did not have long-term follow-up of these survivors, and information regarding their neurodevelopmental outcomes was not available.

## Conclusion

Approximately three-fourths of young infants with acute bacterial meningitis experienced neurological complications despite prompt administration of appropriate and effective antibiotics, which highlights the importance of aggressive follow-up. Most of these patients received conservative treatments. Neurological sequelae were noted in a high percentage of the surviving patients, especially in patients who required surgical interventions. Early occurrence of seizure and septic shock can be a predictor of an unfavorable outcome. Therefore, long-term follow-up of these cases is warranted, as they are at risk of neurodevelopmental impairment.

## Author contributions

M-HH conceptualized and designed the study, drafted the initial manuscript, and approved the final manuscript as submitted. J-FH designed the data collection instruments, and coordinated and supervised data collection and the whole study. H-CK performed the statistical analysis of this study. M-YL helped to collect and verify the data. Y-JL helped to perform the statistical analysis of this study. S-MC performed the microbiological characteristics of this study. H-RH took care of these patients, and carried out the initial analyses. M-CC took care of these patients, and helped data verification. M-HT critically reviewed the manuscript, revised the manuscript, and approved the final manuscript as submitted.

### Conflict of interest statement

The authors declare that the research was conducted in the absence of any commercial or financial relationships that could be construed as a potential conflict of interest.
